# Supplementary material for: A high-resolution mRNA expression time course of embryonic development in zebrafish
Source: eLife. 2017 Nov 16;6:e30860. doi: 10.7554/eLife.30860 (PMC5690287; doi:10.7554/eLife.30860)
Supplement: Supplementary file 6. [file elife-30860-supp6.zip › biolayout-clusters-files/Cluster189-genes.html]

Cluster189


# Cluster189: Genes

| Ensembl ID | Gene Name | Chr | Start | End | Biotype |
| --- | --- | --- | --- | --- | --- |
| ENSDARG00000026967 | ap2s1 | 15 | 8791579 | 8823906 | protein\_coding |
| ENSDARG00000057853 | atp6v0ca | 3 | 15894880 | 15905663 | protein\_coding |
| ENSDARG00000025977 | blmh | 15 | 28701704 | 28852636 | protein\_coding |
| ENSDARG00000059770 | cpt1aa | 7 | 48532758 | 48557561 | protein\_coding |
| ENSDARG00000005451 | gdi2 | 4 | 11465406 | 11478781 | protein\_coding |
| ENSDARG00000101622 | mrps7 | 3 | 11106415 | 11108933 | protein\_coding |
| ENSDARG00000013078 | ywhaba | 6 | 52212575 | 52225290 | protein\_coding |
